# Prevalence of Depression and Anxiety Among High School Teachers During the COVID-19 Pandemic in Riyadh, Saudi Arabia

**DOI:** 10.7759/cureus.51338

**Published:** 2023-12-30

**Authors:** Sulaiman A Alshammari, Sheluweeh M Alenzi, Lamyaa A Alsanad, Shouq A Alhathal, Raghad S Younis, Ghada S Alenazi, Rafeef R Almubarak, Alanoud M Alhudaib

**Affiliations:** 1 Family and Community Medicine, King Saud University, Riyadh, SAU; 2 Child Psychiatry, King Saud University, Riyadh, SAU

**Keywords:** anxiety, covid-19, depression, stress, teachers

## Abstract

Objectives

We aimed to determine the prevalence of anxiety and depression among high school teachers and assess the functionality of teachers with anxiety and depression during the COVID-19 pandemic.

Methods

This cross-sectional survey was conducted during the COVID-19 pandemic. High school teachers participated in the study in Riyadh, Saudi Arabia, between June and December 2022. The online questionnaire barcode was distributed physically to 382 male and female teachers. The questionnaire asked participants to provide demographic data and respond to questions about their feelings of depression and anxiety during the pandemic.

Results

Of the 382 participants, 62.3% were women, 44.2% were aged between 36 and 45 years, 77.5% were married, and 44.2% had 16 years or more of teaching experience. More than two-thirds (68.3%) of the participants were experiencing a moderate level of anxiety, and 73.8% were moderately depressed. The mean depression score (16.76±5.59) was significantly higher for those aged 25-35 (p=0.05). Female teachers scored higher in generalized anxiety disorder (13.83±4.77) than male teachers (12.79±3.89) (p=0.03). Participants with a master's degree had a higher mean score of generalized anxiety disorder (13.75±3.91) (p=0.05). Most subjects overcame the pandemic's psychological effects and coped with their daily routines.

Conclusion

Over half of the participants reported experiencing anxiety or depressive symptoms during the pandemic. However, this research provides policymakers and educators in Saudi Arabia with a unique perspective on a particular geographic area and educational context, which can be of great value. It stresses the need for mental health services in schools to support the well-being of students and teachers. It underscores the significance of addressing mental health concerns among educators during times of crisis. Therefore, school authorities and policymakers should focus on establishing and promoting mental health services during future pandemics.

## Introduction

The COVID-19 outbreak was announced as a pandemic on March 11, 2020 [1]. The Ministry of Health, Saudi Arabia, reported the first case of the pandemic on March 2, 2020, followed by an increasing number of registered cases before the end of the month [2]. Shortly after detecting the primary cases, all schools, events, sports, and travel were suspended in Saudi Arabia on March 8, 2020 [1]. Besides the physiological deterioration caused by the disease, the abrupt change in people's lives negatively affected their mental health [3]. In particular, high school teachers had the additional burden of following precautionary guidelines that restrained them from their normal work obligations and continuously adjusting to the unprecedented idea of online learning [3,4]. These new guidelines caused stress for teachers because they had not been prepared for an emergency like the pandemic [5]. The feeling of instability and the significant need to rapidly switch and adjust to online instruction left teachers feeling overpowered and stressed about the consequences of the pandemic [6,7].

Previous investigations conducted during the pandemic have established that certain groups are more prone to developing psychological distress than others [6], including older adults, women, family members, healthcare workers, and those at risk of disease [8]. Studies have shown that female teachers are more prone to developing psychological effects due to the pandemic than male teachers [7,9]. It has also been reported that teachers who work in private schools experience lower levels of distress than teachers in public schools [6,10]. During the pandemic, teachers were worried about their safety and fearful about the uncertainty of the future, which increased their stress and anxiety levels [6]. We hypothesized that most of the teachers in our study would have considerable symptoms of depression and anxiety, interfering with their everyday routines throughout the pandemic.

However, no study has examined the psychological outcome of the pandemic on Saudi teachers. Therefore, we assessed the symptoms of depression and anxiety suffered by high school teachers in Saudi Arabia throughout the pandemic and the functioning of teachers experiencing depression or anxiety during the pandemic.

## Materials and methods

Participants and procedure

The researchers conducted a cross-sectional investigation in Riyadh, Saudi Arabia, from June to December 2022. First, we divided the Directorate of Education regions of the Riyadh area into five sections: north, east, west, south, and central. Following a simple random technique, we selected four sex-segregated schools from each section, resulting in 10 all-male and 10 all-female schools. After obtaining permission, the investigators approached the selected school leaders and provided them with an online barcode to access a self-administered questionnaire using Google Forms (Google LLC, California, USA). The participants included male and female high school teachers with at least one year of experience. Teachers who already had a psychological diagnosis or were unwilling to participate were excluded.

According to a previous study [3], the average prevalence of anxiety and depression in China was 35%. Thus, assuming a 95% confidence level and a 0.05 error, the calculated sample was 351 participants based on the formula z2p(1-p)/e2. In the event of a 90% response rate, the minimum number of participants needed was 380.

Measures

Three sections comprised the questionnaire. The first section requested demographic information, including age, sex, level of education, marital status, socioeconomic status, and subject taught. Generalized Anxiety Disorder-7 (GAD-7) was administered to evaluate the level of anxiety in the second section. The Patient Health Questionnaire-9 (PHQ-9) was used to measure the degree of depression in the third section. Both GAD-7 and PHQ-9 are valid and reliable instruments [11].

The sensitivity of the GAD-7 is 89%, and its specificity is 82% [12]. The questionnaire consists of seven questions, each scored on a 4-point scale (0-3). There are four possible responses: not at all, several days, more than half of the days, and nearly every day. A score of 10 or greater was deemed an acceptable threshold for identifying anxiety. The range of 0 to 5 points, 6 to 15 points, and 16 or more points indicated mild, moderate, and severe anxiety, respectively [12].

The sensitivity and specificity of the PHQ-9 are 88%. The questionnaire consists of nine items, each scored on a 4-point scale (0-3). There are four possible responses: not at all, several days, more than half of the days, and nearly every day. A score of 10 or higher was deemed an acceptable threshold for identifying depression [13]. The functionality of teachers was assessed by a direct question on whether the teacher's work was affected during the pandemic.

A family physician and a psychiatrist reviewed the questionnaire. The questionnaire was piloted, with 30 teachers excluded from the final analysis. It took approximately seven minutes to complete the questionnaire. Consent was obtained after participants were apprised of the goals of the investigation and their right to opt out at any point without liability to the research group. The data of participants were anonymized.

Ethical approval

The Institutional Review Board of Health Sciences Colleges Research on Human Subjects of King Saud University College of Medicine approved the study (approval number: E-22-7059_F8_CMED).

Data analysis

SPSS Statistics version 26.0 (IBM Corp. Released 2019. IBM SPSS Statistics for Windows, Version 26.0. Armonk, NY: IBM Corp.) was used to analyze the data. Descriptive statistics (frequencies, percentages, means, and standard deviations) characterized the categorical and continuous variables. Pearson's chi-square test was used to examine the associations between categorical variables following a bivariate analysis. Statistical significance was determined using a p-value of 0.05 and a confidence interval of 95%.

## Results

Descriptive statistics

A total of 382 participants voluntarily completed the questionnaire. Table 1 presents the participants' demographic information, living arrangements, and health status. Most (62.3%) participants were female; the most common age group was 36-45 (44.2%). A significant proportion of the participants, namely 77.5%, reported being married, while almost half of them, 49.0%, indicated having more than three children. Regarding level of education, 84.8% had earned a bachelor's degree. The predominant type of school was a public high (75.9%). Half (51.3%) of the participants taught sciences. Participants in the eastern education office represented 40.6% of the sample, and 64.4% of the participants reported satisfaction with the services provided by the education office. Regarding teaching experience, 44% had 16 or more years of teaching experience. Most participants (52.9%) indicated their financial status was good, 95.3% perceived their health as good, and 95.3% did not suffer from mental illness. Almost all (95.3%) of the participants lived with their families.

**Table 1:** Participants’ demographic information, living arrangements, and health status (n=382)

Variable	n	Percent
Gender		
Male	144	37.7
Female	238	62.3
Age		
25–35	89	23.3
36–45	169	44.2
46–55	109	28.5
Above 55	15	3.9
Marital status		
Married	296	77.5
Single	51	13.4
Divorced	26	6.8
Widowed	9	2.4
Number of children		
I don’t have children	70	18.3
1-3	125	32.7
More than 3 children	187	49
Education level		
Bachelor's	324	84.8
Postgraduate	58	15.2
School type		
Public	290	75.9
Private	92	24.1
Subject taught		
Humanitarian	120	31.4
Religious	66	17.3
Scientific	196	51.3
Education office		
South	31	8.1
East	155	40.6
North	99	25.9
West	38	9.9
Middle	59	15.4
Does the educational office provide adequate services?		
Yes	246	64.4
No	136	35.6
Years of teaching experience		
Less than 5	51	13.4
5–15	162	42.4
16 or more	169	44.2
Financial status		
Not sufficient	32	8.4
Good	202	52.9
Excellent	148	38.7
Health status		
Good	364	95.3
Not good	18	4.7
Do you suffer from any mental illness?		
Yes	15	3.9
No	367	96.1
Do you live alone?		
Yes	18	4.7
No	364	95.3

Inferential statistics

The findings about anxiety and depression among the participants are presented in Table 2. The highest prevalence rate was for moderate depression (73.8%) and anxiety (68.3%).

**Table 2:** Prevalence and severity of anxiety and depression symptoms

Symptoms	High n (%)	Moderate n (%)	Low n (%)	Median
Anxiety	42 (11)	261 (68.3)	79 (20.7)	2.00
Depression	21 (5.5)	282 (73.8)	79 (20.7)	2.00

Table 3 presents the mean scores for depression and anxiety by age, sex, and education level.

**Table 3:** Depression and generalized anxiety disorder variation by participants age, sex, and educational level (n=382)

Variable	Age group	n	Mean		p-value
Generalized anxiety disorder	25-35 yrs	89	13.91±4.82		0.19
	36-45 yrs	169	13.57±4.31		
	46-55 yrs	109	13.14±4.58		
	Above 55 yrs	15	11.40±3.04		
Depression	25-35 yrs	89	16.76±5.59		0.05
	36-45 yrs	169	15.37±3.89		
	46-55 yrs	109	15.30±4.87		
	Above 55 yrs	15	14.27±4.89		
Variable	Sex	n		Mean	p-value
Generalized anxiety disorder	Male	144		12.79±3.89	0.03
	Female	238		13.83±4.77	
Depression	Male	144		15.87±4.57	0.97
	Female	238		15.49±4.76	
Variable	Educational level	n		Mean	p-value
Generalized anxiety disorder	Bachelor's	324		13.47±4.56	0.05
	Master's	52		13.75±3.91	
	Ph. D.	6		9.17±2.99	
Depression	Bachelor's	324		15.59±4.74	0.13
	Master's	52		16.29±4.34	
	Ph. D.	6		12.33±3.08	

No statistically significant differences existed in the generalized anxiety disorder mean score by age group. However, the highest mean score of depression was 16.76±5.59 in favor of 25-35 years, which indicates a statistically significant difference (p=0.05).

The female teachers scored 13.83±4.77 in generalized anxiety disorder, higher than their counterpart male teachers (12.79±3.89) (p=0.03). There were no statistically significant differences in the depression mean score by participant sex.

The highest mean of generalized anxiety disorder was 13.75±3.91 in favor of participants with a master's degree (p=0.05). Despite the highest mean score of depression being 16.29±4.34 in favor of participants with a master's degree, there were no statistically significant differences in educational level based on depression.

Table 4 shows the results for the difficulties participants face in work, domestic responsibilities, and interpersonal relationships (functionality) by levels of anxiety and depression, gender, and education level.

**Table 4:** Difficulties participants faced in work, taking care of things at home, or getting along with other people (functionality) by depression level, anxiety, gender, and education level

Variable	Not difficult at all n (%)	Somewhat difficult n (%)	Very difficult n (%)	Extremely difficult n (%)	Chi-square	p-value
Depression						
Low	67	12	0	0	71.196	0.000
	(30.0)	(8.4)	(0.0)	(0.0)		
Moderate	152	120	10	0		
	(68.2)	(83.9)	(66.7)	(0.0)		
High	4	11	5	1		
	(1.8)	(7.7)	(33.3)	(100.0)		
Anxiety						
Low	69	10	0	0	59.148	0.000
	(30.9)	(7.0)	(0.0)	(0.0)		
Moderate	142	110	9	0		
	(63.7)	(76.9)	(60.0)	(0.0)		
High	12	23	6	1		
	(5.4)	(16.1)	(40.0)	(100.0)		
Gender						
Male	85	51	8	0	2.435	0.487
	(38.1)	(35.7)	(53.3)	(0.0)		
Female	138	92	7	1		
	(61.9)	(64.3)	(46.7)	(100.0)		
Experience						
Less than 5 years	24	24	2	1	10.220	0.116
	10.8	(16.8)	(13.3)	(100.0)		
5–15 years	93	62	7	0		
	(41.7)	(43.4)	(46.7)	(0.0)		
16 years or more	106	57	6	0		
	(47.5)	(39.9)	(40.0)	(0.0)		
Total	223	143	15	1		
	100.0%	100.0%	100.0%	100.0%		

Most participants had effective coping mechanisms in dealing with the psychological ramifications and tended to respond with "not difficult at all." However, levels of anxiety and depression were significantly associated with functionality, whereas gender and level of education were not significantly associated with functionality.

## Discussion

During the COVID-19 outbreak, strict preventive measures were implemented, such as "social distancing," "lockdown," and "work from home," for high school teachers in Riyadh, Saudi Arabia. Hence, this study aimed to understand the adverse psychological effects of the pandemic among teachers in Riyadh high schools [14]. Most high school teachers in the research reported high scores on the GAD-7 and PHQ-9, indicating significant levels of anxiety and depression among teachers during the pandemic. Prevalence estimates for depression varied between 5.5% and 73.8%, and anxiety ranged from 11% to 68.3%.

Our findings differ from Li et al.'s [15] study with Chinese teachers. However, this was similar to a web-based survey in Bangladesh [16], whose results found that 35.4% of teachers suffered from depression and 43.7% from anxiety. This also confirms Akour et al.'s [14] findings of a 69.6% prevalence rate of anxiety and depression among university teaching staff in Jordan. Our findings are also similar to those of Silva et al. [17], who found a range of results (from 10% to 49.4%) for the prevalence of anxiety and results (from 15.9% to 28.9%) for depression. Lastly, Ploytabtim et al. [18] found that similarly high rates of anxiety (46.4%) and depression (14.9%) were also seen among teachers at girls' schools in Thailand.

The results of this study revealed sex differences in anxiety among teachers. Female teachers had a significantly higher level of anxiety. However, depression score differences were not significant. Nevertheless, our results differed from Hossain et al. [16], who reported that male teachers were more likely to suffer from depressive symptoms and anxiety than their female counterparts.

In line with the findings of Akour et al., individuals between the ages of 25 and 35 showed the highest mean score for anxiety and depression [13]. However, our findings differed from those of Hossain et al. [16], who found the prevalence of depression and anxiety was lower among younger teachers than their older colleagues. However, the results were consistent with those of Ozamiz-Etxebarria et al. [19], which found a lower level of anxiety in teachers aged 46 and above.

This study showed that the highest mean score for anxiety about the level of education was associated with having a master's degree. This result is similar to the result reported by Santamaria et al. [7], in which most teachers experiencing anxiety were those with a master's degree.

It is evident from the results of this study and other relevant studies that school teachers have psychological issues such as depression and anxiety. During the pandemic, depression and anxiety in high school teachers were associated with their capacity to teach, care for their families, and interact socially.

Teachers may experience various symptoms, such as loss of interest in their work, sleep disturbance, and difficulty in interpersonal relationships. These issues can be alleviated by providing accessible psychological counseling. In some cases, psychiatric intervention may be necessary. Teachers with psychological symptoms may struggle to educate their students, and policymakers may also face challenges in dealing with increased teacher sick leaves and finding substitutes. Therefore, a thorough investigation is needed to understand the extent of these problems and their implications for educators and policymakers.

This study has some strengths. The barcode was distributed physically to ensure a high response rate, thereby reducing selection bias. The study used standardized (PHQ-9) and validated (GAD-7) to measure depression and anxiety. Despite being the first study among schoolteachers in Saudi Arabia, this study has limitations. Because the self-report measures relied on teachers' memories, responses to items may have been affected by recall bias. However, the generalizability of the outcomes may be limited due to the study's exclusive focus on a specific city. Response bias may be introduced due to the self-reported nature of the data, specifically regarding pre-existing mental health conditions; furthermore, the cross-sectional design eliminates the ability to establish causality. Although the research is enlightening, it would benefit from more extensive geographical coverage and a longitudinal methodology.

## Conclusions

The COVID-19 outbreak was associated with elevated levels of depression and anxiety among teachers and other professionals as well. However, most teachers coped well with the circumstances caused by the pandemic and continued to function normally. Mental health services would need to be available/accessible to teachers during crises and catastrophes comparable to the COVID-19 pandemic. Interpersonal, social, and cultural factors should be considered when designing and executing the service. Additionally, to support quality education for students, there is a need to promote mental health services in schools, as they significantly impact the well-being of high school students.

## References

[1] (2023). COVID 19 Public Health Emergency of International Concern (PHEIC). Global research and innovation forum: towards a research roadmap. https://hdl.handle.net/20.500.12663/714.

[2] Alswaidi FM, Assiri AM, Alhaqbani HH, Alalawi MM (2023). Characteristics and outcome of COVID-19 cases in Saudi Arabia: review of six-months of data (March-August 2020). Saudi Pharm J.

[3] Shen X, Yan S, Cao H (2021). Current status and associated factors of depression and anxiety among the Chinese residents during the period of low transmission of COVID-19. Front Psychol.

[4] Aassve A, Alfani G, Gandolfi F, Le Moglie M (2021). Epidemics and trust: the case of the Spanish flu. Health Econ.

[5] Talidong KJB, Toquero CMD (2020). Philippine teachers’ practices to deal with anxiety amid COVID-19. J Loss Trauma.

[6] Allen R, Jerrim J, Sims S (2020). How did the early stages of the COVID-19 pandemic affect teacher wellbeing?. http://chrome-extension://efaidnbmnnnibpcajpcglclefindmkaj/https://repec-cepeo.ucl.ac.uk/cepeow/cepeowp20-15.pdf.

[7] Santamaría MD, Mondragon NI, Santxo NB, Ozamiz-Etxebarria N (2021). Teacher stress, anxiety and depression at the beginning of the academic year during the COVID-19 pandemic. Glob Ment Health (Camb).

[8] Akat M, Karataş K (2020). Psychological effects of COVID-19 pandemic on society and its reflections on education. Turk Stud.

[9] Stachteas P, Stachteas C (2020). The psychological impact of the COVID-19 pandemic on secondary school teachers. Psychiatriki.

[10] Klapproth F, Federkeil L, Heinschke F, Jungmann T (2020). Teachers’ experiences of stress and their coping strategies during COVID-19 induced distance teaching. J Pedagogical Res.

[11] AlHadi AN, AlAteeq DA, Al-Sharif E (2017). An Arabic translation, reliability, and validation of patient health questionnaire in a Saudi sample. Ann Gen Psychiatry.

[12] Spitzer RL, Kroenke K, Williams JB, Löwe B (2006). A brief measure for assessing generalized anxiety disorder: the GAD-7. Arch Intern Med.

[13] Kroenke K, Spitzer RL, Williams JB (2001). The PHQ-9: validity of a brief depression severity measure. J Gen Intern Med.

[14] Akour A, Al-Tammemi AB, Barakat M, Kanj R, Fakhouri HN, Malkawi A, Musleh G (2020). The impact of the COVID-19 pandemic and emergency distance teaching on the psychological status of University Teachers: a cross-sectional study in Jordan. Am J Trop Med Hyg.

[15] Li Q, Miao Y, Zeng X, Tarimo CS, Wu C, Wu J (2020). Prevalence and factors for anxiety during the coronavirus disease 2019 (COVID-19) epidemic among the teachers in China. J Affect Disord.

[16] Hossain MT, Islam MA, Jahan N (2022). Mental health status of teachers during the second wave of the COVID-19 pandemic: a web-based study in Bangladesh. Front Psychiatry.

[17] Silva DF, Cobucci RN, Lima SC, de Andrade FB (2021). Prevalence of anxiety, depression, and stress among teachers during the COVID-19 pandemic: a PRISMA-compliant systematic review. Medicine (Baltimore).

[18] Ploytabtim K, Chandarasiri P, Buathong N, Prasartpornsirichoke J (2021). Prevalence and associated factors of anxiety and depression among teachers at one private all-girls boarding school. J Psychiatr Assoc Thailand.

[19] Ozamiz-Etxebarria N, Berasategi Santxo N, Idoiaga Mondragon N, Dosil Santamaría M (2020). The psychological state of teachers during the COVID-19 crisis: the challenge of returning to face-to-face teaching. Front Psychol.

